# Notes and News

**Published:** 1903-08-29

**Authors:** 


### NOTES AND NEWS.

M. HENRI DUNANT, of Geneva, the founder of the Red Cross Society, has received the honorary degree of Doctor of Medicine at Heidelberg.

AT the time of going to press we learn that the Dr. Sophia Hickman, who disappeared from the Royal Free Hospital, is still undiscovered.

THE Burton-on-Trent Health Committee have decided to prosecute two doctors for failing to notify a case of small-pox at an inn. It is stated that 18 other cases have resulted from this contagion.

WE learn that Dr. Ramsay Smith, the coroner for Adelaide, has received an intimation from the Attorney-General of South Australia, exonerating him from charges of mutilating corpses and withholding parts from burial, of which he was accused, the Attorney-General expressing himself satisfied that the actions out of which the body-snatching scandals arose were actuated in the interests of public justice.

NEW countries usually exhibit much keener interest in the progress of individual members of the community than old ones. Turning over a Canadian medical journal this fact becomes very apparent. In the list of successful candidates at the medical examination of the Toronto Trinity University we find a portrait of the gold medallist of the year, Dr. Brefney Rudolph O'Reilly, and from the notice attaching to the portrait we were interested to note Dr. B. O'Reilly is the eldest son of Dr. Charles O'Reilly, medical superintendent of the Toronto General Hospital. Dr. B. Rudolph O'Reilly, M.D., C.M., has had a distinguished career, having taken first-class marks and a certificate of honour in the final examination for the Fellowship Degree. He has also distinguished himself in athletics. We congratulate Dr. Charles O'Reilly, whose splendid services to the Toronto General Hospital are not only recognised throughout Canada, but by all who have an intimate knowledge of our hospitals and medical schools.

THE Medical Officer of Health for Essex reports several cases of typhoid fever from polluted shellfish drawn from the mud flats off the coast.

THE progress of the epidemic of small-pox at Cambridge seems now to have been arrested. Notifications of new cases have gradually dwindled until they have practically ceased. Since the epidemic was discovered to be small-pox in the middle of July, the number of patients has reached 144.

THE deterioration and present unsatisfactory condition of the Indian Medical Service is the subject of very severe criticism by a writer in a contemporary journal. It is urged that the Service is not up to modern requirements, that it has no head, and that the Service is divided against itself. One objection pointed out seems very real from an outside point of view, and that is that the pay is considerably below that of the Royal Army Medical Corps. There are numerous other reasons given for the unpopularity of the Service. It is to be hoped that, with the powerful advocacy of Sir Michael Foster in the House of Commons, real grievances will be removed and the Indian Medical Service be once more placed in the position of being able to offer attractions to the best candidates procurable.

THE British Association meets this year at Southport, September 9th being the date fixed for the inaugural address of the President, Sir Norman Lockyer. The programme of the sectional meetings offers a formidable list of technical subjects, many of which must of necessity appeal to the expert few. In the Section of Mathematical and Physical Science much interest will attach to a discussion on the nature of the emanations from radio-active substances. This is to be opened by Professor Rutherford, of Montreal, who will give an account of the experiments which have led him to conclude that the emanations from radium are material. The Chemical Section is to receive communications on a great variety of subjects, including Dr. Crossley's researches in the Turpentines and Camphors. Dr. Louis Sambon is to read a paper in the Section of Geography on the Geographical Distribution of Disease and Disease Carriers. The present political situation will add a specially keen interest to the meeting of the Section of Economic Science, in which an entire day has been set aside for the discussion of the fiscal questions that have been raised by Mr. Chamberlain's proposals. The "Professors," as the public prints show, are not by any means unanimous, and we wish them in their meeting a serene and academic calm. "The Shibboleths of Free Trade," which is the title of one of the papers, does not altogether suggest the peace of a happy family. The Engineering Section is to discuss the Problems of Modern Street Traffic—a subject of wide and, to Londoners, of even acute interest. In the Section of Anthropology, Professor Symington, of Belfast, will occupy the chair, and will give an address on various questions associated with the form of the skull. Education in many both of its theoretical and practical aspects will occupy the attention of the Educational Section, and, if recent precedents are to be followed, some lively discussions and novel developments may be expected.

